# Dissecting the role of histidine kinase and HOG1 mitogen-activated protein kinase signalling in stress tolerance and pathogenicity of *Parastagonospora nodorum* on wheat

**DOI:** 10.1099/mic.0.000280

**Published:** 2016-06

**Authors:** Evan John, Francisco Lopez-Ruiz, Kasia Rybak, Carl J. Mousley, Richard P. Oliver, Kar-Chun Tan

**Affiliations:** ^1^​ Department of Environment and Agriculture, Centre for Crop and Disease Management, Curtin University, Bentley, WA 6102, Australia; ^2^​ School of Biomedical Sciences, CHIRI Biosciences Research Precinct and Faculty of Health Sciences, Curtin University, Bentley, WA 6102, Australia

**Keywords:** Hog1, MAPK, histidine kinase, Parastagonospora nodorum, septoria nodorum blotch, 6-methoxy-benzoxazolinone

## Abstract

The HOG1 mitogen-activated protein kinase (MAPK) pathway is activated through two-component histidine kinase (HK) signalling. This pathway was first characterized in the budding yeast *Saccharomyces cerevisiae* as a regulator of osmotolerance. The fungus *Parastagonospora nodorum* is the causal agent of septoria nodorum blotch of wheat. This pathogen uses host-specific effectors in tandem with general pathogenicity mechanisms to carry out its infection process. Genes showing strong sequence homology to *S. cerevisiae* HOG1 signalling pathway genes have been identified in the genome of *P. nodorum*. In this study, we examined the role of the pathway in the virulence of *P. nodorum* on wheat by disrupting putative pathway component genes: HOG1 (SNOG_13296) MAPK and NIK1 (SNOG_11631) hybrid HK. Mutants deleted in NIK1 and HOG1 were insensitive to dicarboximide and phenylpyrrole fungicides, but not a fungicide that targets ergosterol biosynthesis. Furthermore, both Δnik1 and Δhog1 mutants showed increased sensitivity to hyperosmotic stress. However, HOG1, but not NIK1, is required for tolerance to elevated temperatures. HOG1 deletion conferred increased tolerance to 6-methoxy-2-benzoxazolinone, a cereal phytoalexin. This suggests that the HOG1 signalling pathway is not exclusively associated with NIK1. Both Δnik1 and Δhog1 mutants retained the ability to infect and cause necrotic lesions on wheat. However, we observed that the Δhog1 mutation resulted in reduced production of pycnidia, asexual fruiting bodies that facilitate spore dispersal during late infection. Our study demonstrated the overlapping and distinct roles of a HOG1 MAPK and two-component HK signalling in *P. nodorum* growth and pathogenicity.

## Introduction

All organisms adapt to environmental changes through the perception of stimuli. Once the stimulus is recognized, a signal is relayed within the cell, which instigates changes in cellular functions that allow the organism to adapt appropriately. Although fungi inhabit a broad range of environmental niches, numerous signalling pathway components are highly conserved across the kingdom (Bahn* et al.*, 2007). A major area of fungal research in recent years has been dedicated to understanding the intricate mechanisms of cellular adaptation to abiotic stress as perceived during infection of animal and plant hosts. The extent of these adaptations may influence the outcome of an infection in an environment where biotic and abiotic stresses are prevalent. Mitogen-activated protein kinase (MAPK) signalling pathways are some of the best-studied signal transduction pathways to date (Xu, 2000; Zhao* et al.*, 2007). The cascade is activated through an upstream receptor responding to a stimulus. Activation results in the phosphorylation of the MAPK kinase kinases (MAPKKKs), which then phosphorylate the MAPK kinases (MAPKKs). Activated MAPKKs then phosphorylate MAPKs, which regulate gene expression and downstream cellular responses. Five major MAPK pathways have been identified in the budding yeast *Saccharomyces cerevisiae* (Madhani & Fink, 1998). These are the FUS3 (mating), KSS1 (pheromone response/filamentous growth), HOG1 (osmoregulation), SLT2 (cell-wall integrity) and SMK1 (sporulation) pathways. MAPK signalling is implicated in growth, differentiation, survival and pathogenesis in many phytopathogenic filamentous fungi (Xu, 2000; Zhao* et al.*, 2007). They typically contain three major MAPK pathways: orthologues of the FUS3/KSS1, SLT2 and HOG1 MAPK signalling pathways (Xu, 2000).

The HOG1 MAPK pathway was first identified in *S. cerevisiae* as a regulator of osmotolerance (Brewster* et al.*, 1993). Activation of this pathway results in the phosphorylation of Ssk2p/Ste11p MAPKKK, which then phosphorylates the Pbs2p MAPKK. Activated Pbs2p then phosphorylates Hog1(Gustin* et al.*, 1998). Hog1 orthologues in phytopathogenic fungi have a similar role in maintaining osmotolerance (Xu, 2000), but are also involved in other abiotic stress responses, conidiation, pathogenicity, mycotoxin production and susceptibility to dicarboximide/phenylpyrrole fungi cides (Table 1). These phenotypes often vary between different fungal pathogens (Table 1). In *S. cerevisiae*, the HOG1 MAPK signalling pathway is regulated by an upstream two-component sensor-signalling system (Maeda* et al.*, 1994). The sensor/histidine kinase (HK) Sln1p is inhibited by osmotic stress, thereby reducing the level of phosphorylation of the phosphotransferase Ypd1 and response regulator (RR) Ssk1. Together, this leads to an Ssk2p-dependent activation of the HOG1 pathway (Posas* et al.*, 1996). Most fungi contain both the HK and RR domains within the same polypeptide (Grebe & Stock, 1999). Hybrid HKs often utilize an additional round of phosphorylation through a histidine phosphotransferase (Hpt) and a secondary RR. Genome sequencing has revealed that filamentous fungi possess a diverse number of genes that encode putative hybrid HKs. Catlett *et al.* (2003) classified fungal hybrid HKs into 11 major groups. Group III NcNIK1/OS-1 hybrid HKs are involved in osmosensing. Perturbation of group III hybrid HKs in phytopathogens often results in phenotypes that overlap with those of mutants that carry impairment in the HOG1 MAPK cascade (Table 1). However, that this is not always the case. Lin & Chung (2010) demonstrated redundant and distinct roles of AaHOG1 and AaHSK1 (group III HK) in the citrus brown spot pathogen *Alternaria alternata*. Mutants disrupted in AaHSK1 and AaHOG1 demonstrate increased sensitivity to hyperosmotic stress. AaHOG1, but not AaHSK1, is required for virulence on citrus and tolerance to oxidative stress. However, deletion of AaHSK1, but not AaHOG1, conferred resistance to dicarboximide/phenylpyrrole fungicides.

**Table 1.** Characterised functions of putative HK/Hog1 MAPK pathway components in various fungal phytopathogens

**Fungus**	**Gene**	**Family**	**Gene inactivation phenotype**	**Reference**
*Alternaria alternata*	AaHSK1	Group III HK	Δaahog1: non-pathogenic (not Δaahsk1) Δaahsk1: resistant to dicarboximide and phenylpyrrole (not Δaahog1) Δaahog1: increased sensitivity to oxidative stress (not Δaahsk1) Δaahsk1/Δaahog1: increased sensitivity to hyperosmotic stress	Lin & Chung (2010)
	AaHOG1	Hog1 MAPK		
*Alternaria brassicicola*	AbNIK1	Group III HK	Reduced virulence; increased sensitivity to hyperosmotic stress	Avenot* et al.* (2005); Cho* et al.* (2009)
*Alternaria longpipes*	AlHK1	Group III HK	Increased virulence; increased resistance to dimethachlon and phenylpyrrole; reduced sporulation; increased sensitivity to osmotic stress	Luo* et al.* (2012)
*Bipolaris oryzae*	SRM1	Hog1 MAPK	Increased sensitivity to oxidative/UV/hyperosmotic stresses; moderate resistance to dicarboximide and phenylpyrrole; positive regulator of melanin biosynthetic genes	Moriwaki* et al.* (2006)
*Botrytis cinerea*	BcSak1	Hog1 MAPK	Δbcsak1: reduced phytotoxin production Δbos1: increased resistance to dicarboximide and phenylpyrrole (not Δbcos4/Δbcsak1) Δbcos4: reduced glycerol accumulation Δbcsak1Δ/bcos4: abnormal vegetative growth Δbos1/Δbcsak1/Δbcos4: reduced/abolished conidiation Δbos1/Δbcsak1/Δbcos4: non-pathogenic Δbos1/Δbcsak2/Δbcos4: increased sensitivity to oxidative and hyperosmotic stresses	Heller* et al.* (2012); Liu* et al.* (2008); Segmuller* et al.* (2007); Viaud* et al.* (2006); Yang* et al.* (2012)
	BcOS4	Ssk2 MAPKKK		
	Bos1	Group III HK		
*Cochliobolus heterostrophus*	Ssk1	RR	Δssk1: decreased conidiation *in vitro* (not Δhog1) Δhog1: abnormal appressoria formation Δssk1/Δhog1: increased sensitivity to oxidative stress Δssk1/Δhog1/Δdic1: increased sensitivity to hyperosmotic stress Δssk1/Δhog1: reduced virulence Δskn7/Δssk1/Δdic1: increased resistance to dicarboximide and phenylpyrrole	Igbaria* et al.* (2008); Izumitsu* et al.* (2007); Oide* et al.* (2010); Yoshimi* et al.* (2004); Yoshimi* et al.* (2005)
	Skn7	RR		
	Hog1	Hog1 MAPK		
	Dic1	Group III HK		
*Colletotrichum lagenarium*	OSC1	Hog1 MAPK	Increased sensitivity to hyperosmotic stress; increased resistance to phenylpyrrole	Kojima* et al.* (2004)
*Cryphonectria parasitica*	CpMK1	Hog1 MAPK	Increased sensitivity to hyperosmotic stress; reduced virulence; reduced conidiation; reduced pigmentation	Park* et al.* (2004)
*Fusarium graminearum*	FgOS2	Hog1 MAPK	Δfgos2/Δfghog1/Δfgpbs2/Δfgssk2: increased sensitivity to oxidative and hyperosmotic stresses Δfghog1/Δfgpbs2/Δfgssk2: cell-wall defect Δfgos2/Δfghog1/Δfgpbs2/Δfgssk2: reduced virulence Δfghog1/Δfgpbs2/Δfgssk2: vegetative growth defect Δfgos2: increased resistance to phenylpyrrole; reduced production of deoxynivalenol and zealerone in planta; inability to produce ascospores Δfghog1: reduced accumulation of glycerol, mannitol, arabitol and sucrose	Nyugen* et al.* (2012); Zheng* et al.* (2012)
	Fghog1	Hog1 MAPK		
	Fgpbs2	Pbs2 MAPKK		
	Fgssk2	Ssk2 MAPKKK		
*Fusarium oxysporum*	Fhk1	Group III HK	Increased resistance to dicarboximide and phenylpyrrole; increased sensitivity to hyperosmotic and oxidative stresses; reduced virulence	Rispail & Di Pietro (2010)
*Fusarium proliferatum*	Fphog1	Hog1 MAPK	Increased resistance to phenylpyrrole; increased sensitivity to oxidative, heat, UV and hyperosmotic stresses	Adam* et al.* (2008a, b)
*Gibberella zeae*	Ssk1	RR	Increased sensitivity to oxidative stress; increased sensitivity to hyperosmotic stress; decreased conidiation; decreased virulence; hydrophillic; delayed ascospore release	Oide* et al.* (2010)
	Hog1	Hog1 MAPK		
*Magnaporthe oryzae*	OSM1	Hog1 MAPK	Δosm1: reduction in arabitol content Δhik1: increased resistance to dicarboximide, phenylpyrrole (Δhik1/Δssk1) and pentachloronitrobenzene Δmossk1: reduced virulence Δmosln1: increased sensitivity to oxidative stress; increased sensitivity to heavy metal; cell-wall defect; non-pathogenic Δmosln1/Δhik1: increased sensitivity to heat stress Δmosln1/Δosm1/Δhik1: increased sensitivity to hyperosmotic stress	Dixon* et al.* (1999); Jacob* et al.* (2014); Motoyama* et al.* (2005, 2008); Zhang* et al.* (2010)
	HIK1	Group III HK		
	MoSLN1	Group VI HK		
	MoSSK1	RR		
*Mycosphaerella graminicola*	MgHog1	Hog1 MAPK	Unable to form filamentous growth/non-pathogenic; impaired pigmentation; increased resistance to dicarboximide and phenylpyrrole; increased sensitivity to hyperosmotic stress	Mehrabi* et al.* (2006)
*Sclerotinia sclerotiorum*	Shk1	Group III HK	Increased resistance to dicarboximide and phenylpyrrole; increased sensitivity to hyperosmotic and oxidative stresses; altered vegetative growth and unable to produce sclerotia	Duan* et al.* (2013)

*Parastagonospora nodorum* is the causal agent of septoria nodorum blotch on wheat (Solomon* et al.*, 2006a; Quaedvlieg* et al.*, 2013). Septoria nodorum blotch is a major disease in many wheat-growing regions of the world (Oliver* et al.*, 2012). *P. nodorum* is a sophisticated fungal necrotroph that utilizes an array of host-specific necrotrophic effectors to manipulate the plant metabolism to favour infection (Friesen* et al.*, 2008; Tan* et al.*, 2010, 2015). The role of the FUS3/KSS1 MAPK orthologue MAK2 in pathogenicity has been examined in detail in *P. nodorum* (Solomon* et al.*, 2006a; Tan* et al.*, 2009b). Targeted gene deletion of *MAK2* resulted in mutants that were non-pathogenic, unable to sporulate and exhibited abnormal vegetative growth (Solomon* et al.*, 2005b). Subsequent proteomic analyses identified a Mak2/Gna1 Gα subunit co-regulated putative short-chain dehydrogenase that plays a role in sporulation and mycotoxin production (Tan, 2007; Tan* et al.*, 2008, 2009c). The function of HOG1 signalling during *P. nodorum* pathogenicity and development is poorly understood. Genome sequencing of *P. nodorum *has revealed genes that encode putative components of the HOG1 pathway (Hane* et al.*, 2007). We decided to investigate the role of the pathway by inactivating components in *P. nodorum* that are orthologous to genes of other fungi known for their overlapping involvement in osmotolerance and susceptibility to dicarboximide/phenylpyrrole fungicides. As such, genes that encode putative group III HK and HOG1 MAPK were knocked out using targeted gene deletion. The resulting mutants were examined for their roles in stress tolerance, development and pathogenicity on wheat. This approach allows a simultaneous comparison of phenotypic effects that were incurred from mutations in different parts of the HOG1 MAPK signalling pathway in *P. nodorum* in order to elucidate overlapping and/or distinct roles of both components.

## Methods

### Fungal culturing.

All* P. nodorum* strains were maintained on V8-PDA agar (150 ml Campbell’s V8 juice  l^−1^, 3 g CaCO_3_ l^−1^, 30 g sucrose  l^−1^ , 10 g Difco PDA l^−1^ and 10 g agar l^−1^) at 21 °C under a 12 h photoperiod.

### *In planta* gene expression analysis.

RNA isolation and *in planta* gene expression analysis were performed as previously described with minor modifications (Solomon* et al.*, 2003). Briefly, detached wheat leaves (Halberd) maintained in 0.15 % (w/v) benzimidazole agar were inoculated with 1×10^6^ pycnidiospores in 0.02 % Tween 20 to facilitate infection. Lesions were excised at 3, 6, 8 and 10 days post-infection (dpi), freeze dried and subject to RNA extraction using TRIzol reagent (Invitrogen), DNase-treated and reverse transcribed as previously described (Tan* et al.*, 2008). Quantitative real-time PCR (qRT-PCR) was performed using a Quantitect SYBR Green RT-PCR kit (Qiagen) and a Bio-Rad CFX96 system, using SN15 genomic DNA as a quantitative standard. The primer pair Nik1qPCRf and Nik1qPCRr was used to amplify a 129 bp region of NIK1. The primer pair Hog1qPCRf and Hog1qPCRr was used to amplify a 144 bp region of HOG1. The housekeeping gene actin (ACT1) was used to normalize gene expression using the primer pair ActinqPCRf and ActinqPCRf (Tan* et al.*, 2008). Primer sequences are shown in Table S1 (available in the online Supplementary Material).

### Construction of the HOG1 and NIK1 gene knockout vectors.

*P. nodorum* SN15 strains carrying individual deletions in HOG1 (HOG1 MAPK gene) and NIK1 (HK gene) were created through genetic transformation using gene knockout vectors generated from fusion PCR (Solomon* et al.*, 2006b) (Fig. S1, available in the online SupplementaryMaterial). For HOG1, 5_Hog1F and 5_Hog1R were used to amplify a 701 bp 5′ UTR (untranslated region) fragment. This was fused to a phleomycin-resistance cassette (BLE) amplified from pAN8-1 using pAN8f and pAN8r. The resulting construct was subsequently fused to an 865 bp 3′ UTR fragment of HOG1 generated using 3_Hog1F and 3_Hog1R (Fig. S1a). For NIK1, 5_Nik1F and 5_Nik1R were used to amplify a 751 bp 5′ UTR fragment. This was fused to a BLE. The resulting construct was subsequently fused to an 814 bp 3′ UTR fragment of NIK1 generated using 3_Nik1F and 3_Nik1R (Fig. S1b). All PCR amplifications were performed with Phusion Taq DNA polymerase (New England Biolabs). Primer sequences for vector construction are shown in Table S1. Gene knockout vectors were subsequently used to transform *P. nodorum* SN15 using PEG-mediated transformation (Solomon* et al.*, 2004). Mutants that carried the appropriate gene deletion were identified using PCR (Fig. S1c). A robust quantitative PCR method was used to determine the copy number of HOG1 and NIK1 deletion constructs in all transformants to identify appropriate mutants with single copy integration (Fig. S1c) (Solomon* et al.*, 2008). Gene deletion mutants and an ectopic strain (Ect) with single copy integration were retained for further studies. All fungal strains used in this study are described in Table 2.

**Fig. 1.**
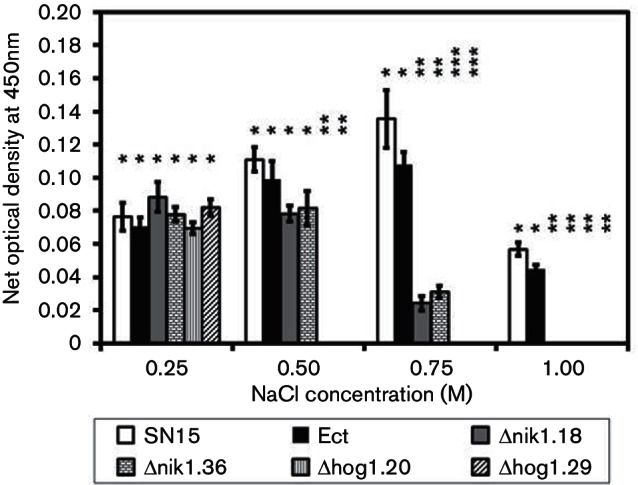
HOG1 and NIK1 are required for osmotolerance. Net optical density was determined following 6 days growth in MM broth supplemented with different NaCl concentrations using a 96-well plate assay as described by Lowe *et al.* (2008). The experiment was performed in four biological replicates. The error bars show sems. ANOVA using the Tukey–Kramer test set at a significance threshold of *P*<0.05 was used to compare all measurements at each NaCl treatment. When indicated by the same number of asterisks above the bars, the mean was not found to be significantly different between strains in each treatment.

**Table 2.** Fungal strains used in this study

**Strain**	**Description**	**Source**
SN15	Wild-type	Department of Agriculture and Food Western Australia, Western Australia, Australia
Ect	Ectopic transformant (BLE ectopic integration)	This study
Δnik1.18	SN15 deleted in SNOG_11631 (NIK1)	This study
Δnik1.36	SN15 deleted in SNOG_11631 (NIK1)	This study
Δhog1.20	SN15 deleted in SNOG_13296 (HOG1)	This study
Δhog1.29	SN15 deleted in SNOG_13296 (HOG1)	This study
Δnik1 :: NIK1	Δnik1.18 complemented with SNOG_11631 (NIK1)	This study
Δhog1 :: HOG1	Δhog1.20 complemented with SNOG_13296 (HOG1)	This study

### Genetic complementation.

*P. nodorum* Δhog1.20 and Δnik1.18 were selected for genetic complementation. For HOG1, a 3931 bp region containing the gene, native promoter and terminator regions was amplified using Hog1FC and Hog1RC. The resulting DNA fragment was fused to a hygromycin resistance gene (HPH) derived from pAN7-1 using fusion PCR (Fig. S2a). For NIK1, a 6317 bp region containing the gene, native promoter and terminator regions was amplified using Nik1FC and Nik1RC. The resulting DNA fragment was fused to HPH (Fig. S2a). All PCR amplifications were performed with Phusion Taq DNA polymerase. Primer sequences for vector construction are shown in Table S1. Genetic complementation vectors were used to transform the respective gene deletion mutants using PEG-mediated transformation (Solomon* et al.*, 2004). Genetically complemented Δhog1 :: HOG1 and Δnik1 :: NIK1 strains were retained for phenotypic analysis.

**Fig. 2.**
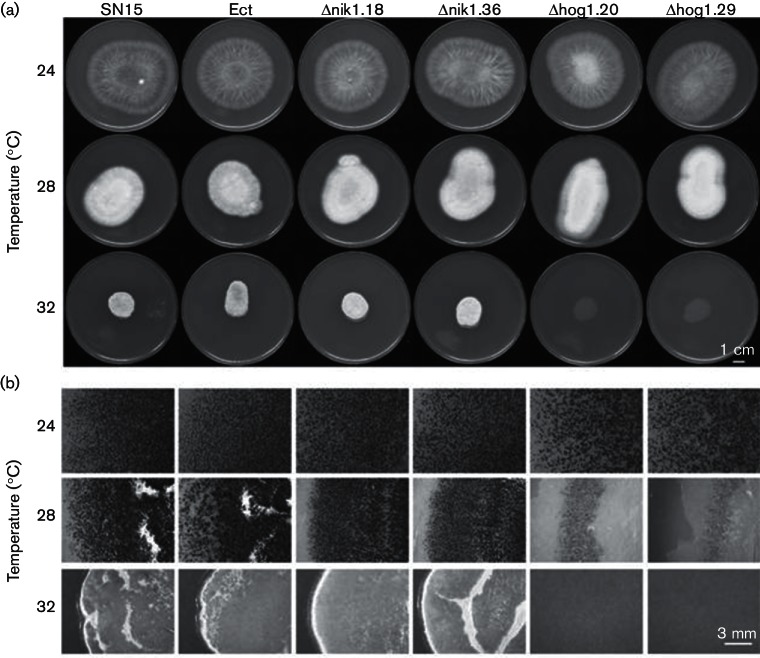
Growth of all strains at elevated temperatures. (a) Colony morphology and (b) pycnidia development were examined.

### Fungicide tolerance assay.

A 96-well microtitre plate assay was used to assess fungicide resistance (Thind, 2011; Weber & Hahn, 2011). Four fungicides from three classes were tested: two dicarboximides (iprodione and procymidone), a phenylpyrrole (fludioxonil) and a triazole (tebuconazole) (Sigma-Aldrich). With each strain, 5 µl spores (10^6^ spores ml^−1^) for the respective mutants/controls was added to minimal medium (MM) broth for a final volume of 100 µl per well (Solomon* et al.*, 2004). Each well was supplemented with a fungicide pre-dissolved (in ethanol) with concentrations ranging from 0 µg ml^−1^ (solvent only) to the maximum workable for the respective fungicides. Growth was measured as the change in optical density at a wavelength of 450 nm from the initial measurement to the final measurement at day 6 post-inoculation using a Synergy HT multi-detection microplate reader (Bio-Tek). Plates were wrapped in parafilm to maintain sterility and incubated at 22 °C in the dark. Measurements were taken as the average of nine readings across the area of each well. The experiment was performed with four biological replicates. For each, regression lines were calculated from plots of log(fungicide concentration) versus log(% inhibition). This allowed an estimation of EC_50_ values (fungicide concentration where growth is inhibited by 50 %), which were used to calculate the resistance factor (RF) for Δhog1/nik1 mutants relative to SN15. Where inhibition of growth was not observed for a particular strain, the highest concentration of fungicide tested was used for calculating this value with resulting RF values reported as ‘>*x*’ where *x* represents the RF.

### Hyperosmotic and oxidative stress tolerance assays.

The ability of the mutants to grow under hyperosmotic and oxidative stress conditions was assessed through a microtitre assay set up essentially as described above, but without fungicides. Following Lowe *et al.* (2008), 0.25, 0.50, 0.75 and 1.00 M NaCl was used for the hyperosmotic stress assay. For the oxidative stress microtitre assay, all *P. nodorum* strains were inoculated with 0, 1, 3 and 10 mM H_2_O_2_.

### 6-Methoxy-2-benzoxazolinone (MBOA) tolerance assay.

A growth assay was employed to determine the impact of MBOA on the growth of all strains. MM agar plates were supplemented with 0, 0.1, 0.3, 1.0 and 3.0 mM MBOA (Sigma-Aldrich) dissolved in 0.4 % (v/v) ethanol (Du Fall & Solomon, 2013). Following this, plates were inoculated with 1×10^4^ spores. The impact of MBOA on fungal growth was determined by measuring colony diameters following 9 days growth at 22 °C under fluorescent light on a 12 h light/dark cycle. The assay was performed in biological triplicates.

### Whole plant spray infection assay.

Virulence was determined using a whole plant spray method as previously described, with minor modifications (Solomon* et al.*, 2005a). Briefly, an inoculum consisting of 1×10^6^ spores ml^−1^ in 0.5 % gelatin (Sigma-Aldrich) was sprayed onto 2-week-old wheat seedlings (Calingiri; InterGrain) via an airbrush system. Disease was allowed to develop in a growth chamber at 22 °C on a 12 h light/dark cycle for 10 days under high humidity prior to scoring. For the whole plant spray assay at an elevated temperature, infection was allowed to develop for 5 days at 22 °C on a 12 h light/dark cycle under high humidity. A temperature cycle of 28 °C for 8 h, followed by 22 °C for 16 h, on a 12 h light/dark cycle under high humidity for 9 days, was used. The first 8 h of the light cycle coincided with the elevated temperature. Following the whole plant spray assessment, five infected first leaves representing each infection were removed and maintained on 0.15 % benzimidazole agar as detached leaves for 14 days for infection at 22 °C and 5 days for the infection at alternating 22 and 28 °C (Solomon* et al.*, 2004). This allowed for an assessment of pycnidiation *in planta*. Images representing the extent of pycnidia formation were taken with a Nikon SMZ 800 stereoscope coupled to a DS-L3 controller.

## Results

### Identification of the putative HOG1 MAPK and NcNIK1/OS-1 class HK in *P. nodorum*

The Hog1 MAPK protein sequence from *S. cerevisiae* (Genbank accession no. CAA97680) was used to search the ‘*Phaeosphaeria nodorum* SN15' Genbank nr protein database and identified SNOG_13296 (Genbank accession no. Q0U4L8) as the best blast hit. The predicted SNOG_13296 polypeptide sequence consisted of 355 aa encoded by a 1475 bp gene comprising seven exons and six introns. Conserve domain (CD)-blast identified a protein kinase domain (pfam00069) from amino acid 20 to 299 (Fig. S3a). Using the filamentous fungal MAPK classification nomenclature described by Xu (2000), phylogenetic analysis classifies the SNOG_13296 polypeptide to be a genuine Hog1 MAPK orthologue (Fig. S4). Amino acid identity of the SNOG_13296 polypeptide to characterized Hog1 MAPKs of fungal phytopathogens ranged from 91 % with Osm1 of the rice blast fungus *Magnaporthe oryzae* (Dixon* et al.*, 1999) to 97 % with Hog1 of the southern corn blight pathogen *Cochliobolus heterostrophus* (Yoshimi* et al.*, 2005).

**Table 3.** EC_50_ (μg ml^−1^) and RF values of the fungicide tolerance assay

**Strain**	**Iprodione**		**Procymidone**		**Fludioxonil**		**Tebuconazole**	
	**EC_50_**	**RF vs SN15**	**EC_50_**	**RF vs SN15**	**EC_50_**	**RF vs SN15**	**EC_50_**	**RF vs SN15**
SN15	1.84	1.00	2.06	1.00	0.10	1.00	1.38	1.00
Ect	1.66	0.90	2.15	1.04	0.20	1.98	1.28	0.93
Δhog1.20	10*	>5.44	10*	>4.86	5*	>48.77	1.66	1.21
Δnik1.18	10*	>5.44	10*	>4.86	5*	>48.77	2.07	1.50
Δhog1 :: HOG1	1.41	0.76	1.55	0.76	0.80	0.74	–	–
Δnik1 :: NIK1	0.63	0.17	0.48	0.23	0.01	0.05	–	–

*EC_50_ is greater than the measurable concentration used; therefore, the highest concentration of fungicide tested was used for RF calculation.

The microtitre growth curves used for calculation.

**Fig. 4.**
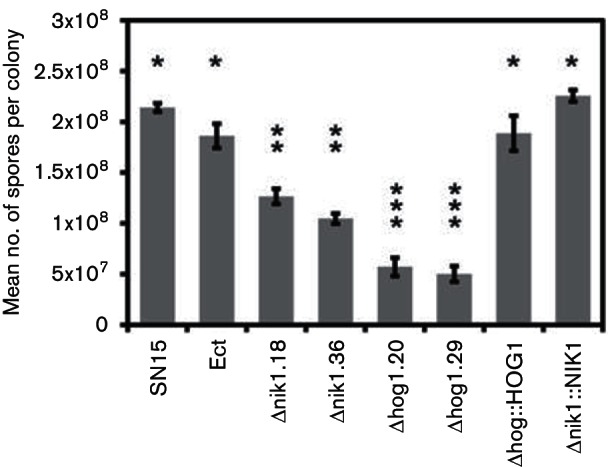
*In vitro* sporulation assay on V8-PDA. ANOVA using the Tukey–Kramer test set at a significance threshold of *P*<0.05 was used to compare sporulation of all strains. The error bars show sems. When indicated by the same number of asterisks above the bars, the mean was not found to be significantly different between strains.

The group III hybrid HK NcNik1/OS-1 from *Neurospora crassa* (Broad Institute accession no. NCU02815.7) identified SNOG_11631 (Genbank accession no. XP_001801869) as the best blast hit. The corrected ORF and amino acid sequence of SNOG_11631 are located at: https://github.com/robsyme/Parastagonospora_nodorum_SN15. CD-blast analysis identified four pfam domains: a HAMP domain (pfam00672) from amino acid 286 to 630, a phospho-acceptor domain from amino acid 740 to 804 (pfam00512), an HK-like ATPase domain (pfam02518) from amino acid 851 to 965, and a RR domain from amino acid 1117 to 1232 (pfam00072) (Fig. S3b). The domain prediction of SNOG_11631 is consistent with that of the group III hybrid HKs (Catlett* et al.*, 2003). Using the fungal hybrid HK classification nomenclature proposed by Catlett *et al.* (2003), phylogenetic analysis further classified the SNOG_11631 polypeptide to be a genuine group III hybrid HK (Fig. S5). Amino acid identity of the SNOG_11631 polypeptide to characterized group III hybrid HKs ranged from 66 % for the grey mould *Botrytis cinerea* (Liu* et al.*, 2008) to 88 % for *C. heterostrophus* (Yoshimi* et al.*, 2004). From here, SNOG_13296 and the revised SNOG_11631 genes are referred to as HOG1 and NIK1, respectively.

**Fig. 5.**
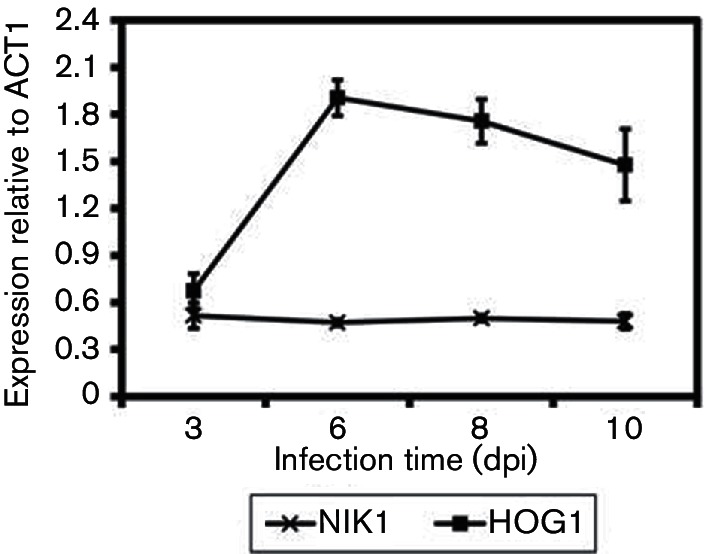
The expression profile of NIK1 and HOG1 *in planta*. The error bars show sems. The experiment was performed in biological triplicates.

### Disruption of NIK1 and HOG1 in *P. nodorum*

To determine the role of the HK-Hog1 MAPK pathway in *P. nodorum* development and pathogenicity, we inactivated NIK1 and HOG1 in the wild-type strain SN15 using targeted gene deletion. Consequently, strains deleted in NIK1 (Δnik1.18 and Δnik1.36), HOG1 (Δhog1.20 and Δhog1.29) and an ectopic (Ect) mutant were retained for phenotypic characterization (Fig. S1, Table 2). The Ect mutant contained a selectable marker insertion elsewhere in the genome rather than in NIK1 and HOG1; thus, all assayed phenotypes should be similar to SN15.

### HOG1 and NIK1 play a role in osmotic stress tolerance

Reduction in osmotic stress tolerance is a hallmark of perturbation in the Hog1 MAPK signalling pathway (Table 1). The osmotolerance of *P. nodorum* Hog1 MAPK pathway mutants was assessed by measuring growth in NaCl concentrations ranging from 0.25 to 1 M, as described by Lowe *et al.* (2008), in a microtitre assay (Fig. 1). To our surprise, SN15 and Ect exhibited a slight increase in growth between 0.25 and 0.75 M NaCl. All strains exhibited similar growth at 0.25 M NaCl. The growth of Δhog1.20 and Δhog1.29 was inhibited at 0.5 M, whereas growth of Δnik1.18 and Δnik1.36 was significantly reduced at 0.75 M. No growth of Δhog1.20, Δhog1.29, Δnik1.18 and Δnik1.36 was seen at 1 M NaCl, while both SN15 and Ect grew to some extent. The results indicate that NIK1 and HOG1 are required for osmotolerance (Fig. 1).

### HOG1 and NIK1 are dispensable for tolerance to oxidative stress

Inactivation of the fungal Hog1 MAPK signalling pathway often resulted in increased sensitivity to oxidative stress (Table 1). The ability of *P. nodorum* Hog1 pathway mutants to tolerate oxidative stress was assessed by measuring growth in 0 to 1 mM H_2_O_2_ in a microtitre assay (Fig. S6). Growth of *P. nodorum* SN15 was observed at 0 and 1 mM H_2_O_2_, but was greatly inhibited at 3 mM and 10 mM H_2_O_2_. *P. nodorum* Δhog1 and Δnik1 strains did not exhibit increased sensitivity to H_2_O_2_ compared to strains with intact HOG1 and NIK1 (Fig. S6).

**Fig. 6.**
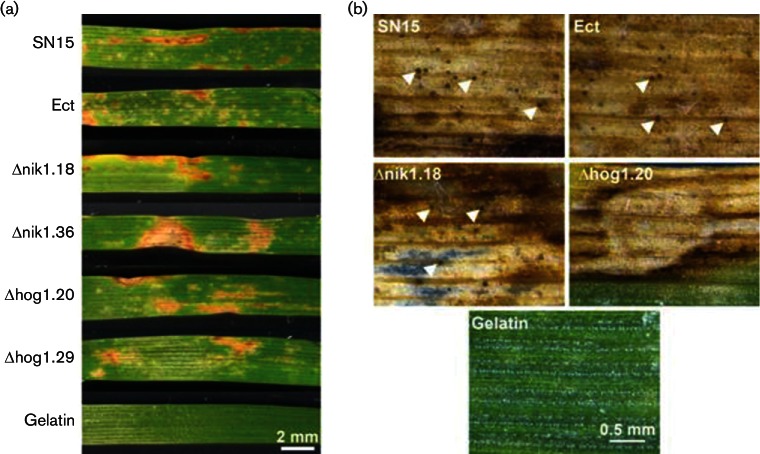
Wheat infection assay at 22 °C. (a) Necrotic lesions caused by SN15, Ect, Δnik1 and Δhog1 mutants. Gelatin was used as a non-infection treatment. (b) Pycnidia were allowed to develop on a detached leaf assay. Arrowheads indicate pycnidia.

### Δnik1 and Δhog1 mutants showed increased resistance to dicarboximide and phenylpyrrole fungicides

Fungal strains perturbed in HOG1 signalling often exhibit resistance to dicarboximide (e.g. iprodione and procymidone) and phenylpyrrole (e.g. fludioxonil) fungicides (Table 1). Microtitre plate growth rates of SN15, Ect, Δhog1.20, Δnik1.18 and complemented strains on various concentrations of iprodione, procymidone, fludioxonil and a control fungicide, tebuconozole, were determined (Table 3, Fig. S7). Both Δhog1.20 and Δnik1.18 displayed high RF values to both dicarboximide and phenylpyrrole fungicides. The EC_50 _values of the deletion mutants were higher than the maximum concentration of iprodione and procymidone used (10 µg ml^−1^). Similarly, the EC_50_ values of the deletion mutants were higher than the maximum concentration of fludioxonil used (5 µg ml^−1^). We then genetically complemented Δhog1.20 and Δnik1.18. Genetic complementation restored sensitivity to iprodione, procymidone and fludioxonil. No resistance to tebuconazole was observed (Table 3, Fig. S7). This indicates that dicarboximide and phenylpyrrole fungicides inappropriately activate the HOG1 pathway in *P. nodorum*.

### HOG1 deletion limits *P. nodorum* growth and pycnidiation at elevated temperatures *in vitro*

During field infection, it is likely that *P. nodorum* experiences changes in the environment, such as temperature fluctuations, and must adapt. The effect of elevated temperatures was investigated to explore further abiotic stress responses associated with NIK1 and HOG1 deletion in *P. nodorum*. Mycelial growth was compared following incubation at 24, 28 and 32 °C for 10 days on V8-PDA agar medium. Vegetative growth of all strains was similar at 24 and 28 °C. However, *P. nodorum* Δhog1.20 and Δhog1.29 were unable to grow at 32 °C (Fig. 2a). Furthermore, hypersensitivity to heat stress in the Δhog1 background was already apparent at 28 °C, as pycnidial development was sparse when compared to SN15 (Fig. 2b). Growth and pycnidiation of the Δnik1 mutants were similar to SN15 (Fig. 2). This indicates that HOG1, but not NIK1, is required for tolerance to heat stress *in vitro*. Genetic complementation of Δhog1.20 restored the SN15-like vegetative growth and pycnidiation (Fig. S8).

### Deletion of HOG1 increases tolerance to the cereal phytoalexin MBOA

**Fig. 3.**
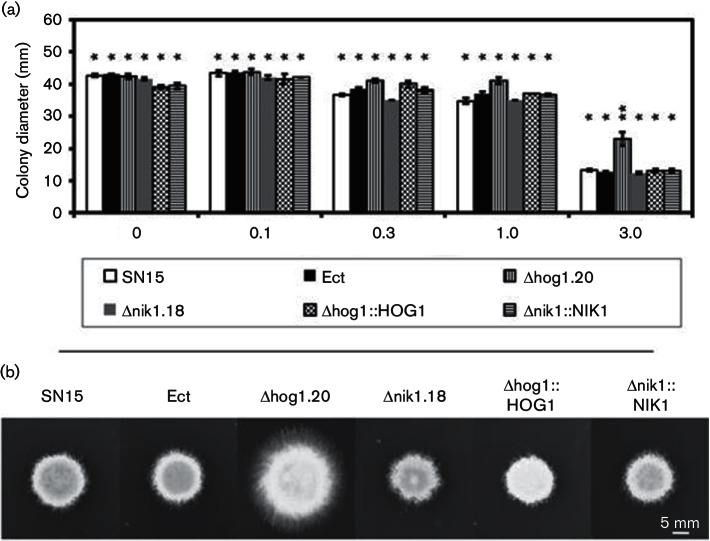
Growth of all strains on MBOA. (a) Colony diameter was determined from 9 days growth on MM agar supplemented with different MBOA concentrations. The experiment was performed in biological triplicates. ANOVA using the Tukey–Kramer test set at a stringent significance threshold of *P*<0.0005 was used to compare the radial growth of all strains. The error bars show sems. When indicated by the same number of asterisks above the bars, the mean was not found to be significantly different between strains in each treatment. (b) Colony morphology of all strains grown on 3.0 mM MBOA.

The cereal phytoalexin MBOA inhibits the growth of *P. nodorum* at high concentrations (Du Fall & Solomon, 2013). A petri dish assay was used to determine the radial growth of SN15, Ect, Δhog1.20, Δnik1.18 and genetically complemented Δhog1 :: HOG1 and Δnik1 :: NIK1 in the presence of MBOA at different concentrations (Fig. 3a). Surprisingly, Δhog1.20, but not Δnik1.18, demonstrated increased tolerance to MBOA at 3.0 mM (Fig. 3b).

### NIK1 and HOG1 deletion reduces asexual sporulation *in vitro*

*P*. *nodorum* produces asexual fruiting bodies called pycnidia. These fruiting bodies contain pycnidiospores, which are required by the fungus to facilitate secondary host infection (Eyal* et al.*, 1987). We sought to investigate for evidence of fitness reduction of pycnidia in the Δhog1 and Δnik1 mutants by quantifying the level of asexual sporulation *in vitro* (Fig. 4). All mutants produced significantly fewer pycnidiospores than SN15. *P. nodorum* Δhog1.20 and Δhog1.29 produced significantly less spores than Δnik1.18 and Δnik1.36.

### HOG1 but not NIK1 is required for full pycnidiation *in planta*

*In vitro* characterization of Δhog1 and Δnik1 mutants has identified the corresponding genes, HOG1 in particular, to be important in *P. nodorum* development and stress tolerance. With this in mind, we sought to investigate expression of HOG1 and NIK1 in SN15 during infection using qRT-PCR. Time points reflecting early infection/host penetration (3 dpi), proliferation (6 dpi) and asexual sporulation/pycnidiation (8 and 10 dpi) were chosen (Fig. 5). The level of NIK1 expression was found to remain constant through the sampled infection period. In contrast, HOG1 expression increased approximately threefold during proliferation, leading to the onset of pycnidiation. Since NIK1 and HOG1 are expressed *in planta*, we then assessed whether these genes play a role in the infection lifecycle of *P. nodorum* on wheat using a whole plant spray assay at 22 °C. It was observed that *P. nodorum* Δhog1.20, Δhog1.29, Δnik1.18 and Δnik1.36 caused necrotic lesions on wheat that were comparable to SN15 (Fig. 6a). However, HOG1 deletion abolished pycnidiation and consequently asexual sporulation *in planta* (Fig. 6b). This suggests that HOG1, but not NIK1, is required to complete asexual development through pycnidiation during wheat infection.

As HOG1 is required for tolerance to elevated temperatures *in vitro*, a whole plant spray assay was undertaken at an elevated temperature to determine whether heat stress affects the virulence of the Δhog1 mutants. A temperature of 28 °C was chosen for infection, as Δhog1 mutants first showed a compromised heat-stress-related phenotype at this temperature. *P. nodorum* Δhog1.20 was able to cause lesions on the host similar to SN15 (Fig. S9a). Surprisingly, Δhog1.20 was able to produce pycnidia *in planta* at 28 °C, unlike the 22 °C infection (Fig. S9b), but at a much reduced number (Fig. S9c). Thus, increased temperature partially complements the pycnidiation defect of Δhog1.20 during growth *in planta*.

## Discussion

Thus far, the FUS3/KSS1 MAPK class MAK2 is the only MAPK gene that has been characterized in *P. nodorum* for its role in virulence and downstream target regulation (Solomon* et al.*, 2005b; Tan, 2007). Analysis of the *P. nodorum* genome identified two other genes that encode putative MAPKs. One of these is HOG1*/*SNOG_13296, which belongs to the HOG1 class. Analysis of the *P. nodorum* genome has also uncovered genes that encode amino acid sequence orthologues to yeast Ssk1 MAPKKK (SNOG_09580), Ssk2 MAPKK (SNOG_07196), Ste11 MAPKK (SNOG_07664) and Pbs2 MAPKK (SNOG_02007). We have also identified a putative SLT2 MAPK class gene, SNOG_05764, which encodes a 420 aa polypeptide (Fig. S4). SNOG_05764 is referred to as MPS1. The role of MPS1 in phytopathogenicity is currently being investigated in our laboratory.

Analysis of the *P. nodorum* SN15 genome identified a large family of potential two-component systems that consisted of 23 HKs, 3 RRs and 1 Hpt (Hane* et al.*, 2007). Analysis of the *C. heterostrophus* genome identified 21 HKs, 3 RRs and 1 Hpt, whereas *N. crassa* possesses 11 HKs, 2 RRs and 1 Hpt (Catlett* et al.*, 2003). Catlett *et al.* (2003) identified 11 major groups based on phylogenetic analysis of predicted hybrid HKs from *C. heterostrophus, N. crassa, B. cinerea* and *Fusarium verticillioides*. All 11 groups possess predicted phospho-acceptor, ATPase and response-regulator domains. Phylogenetic analysis placed the predicted *P. nodorum* hybrid HKs into the 11 major groups (Fig. S5). NIK1/SNOG_11631 is the only *P. nodorum* gene classified within the group III hybrid HK.

HK signalling is often transduced via the Hog1 MAPK pathway in most eukaryotes. In many fungi, perturbation in group III HK and Hog1 often results in overlapping phenotypes, such as a loss in osmotolerance and increased resistance to dicarboximide and phenylpyrrole fungicides (Table 1). Therefore, we hypothesized that Nik1 function in close association with the Hog1 MAPK pathway in *P. nodorum*. We have generated *P. nodorum* strains that carry gene deletions of two putative components of the HOG1 pathway: a putative group III hybrid HK Nik1 and the Hog1 class MAPK Nik1. Mutants carrying deletions in NIK1 and HOG1 showed increased resistance to both dicarboximide and phenylpyrrole antifungal agents consistent with many other fungal pathogens (Table 1). Phenotypic defects relating to asexual sporulation and hyperosmotic stresses were significantly more pronounced in the Δhog1 than the Δnik1 mutants. Furthermore, Δhog1 mutants, but not Δnik1 mutants, displayed increased sensitivity to heat stress. This study has demonstrated both distinct and overlapping roles of group III HK and HOG1 MAPK in *P. nodorum* that are somewhat similar to the phenomenon described by Lin & Chung (2010) for AaHSK1 and AaHOG1 deletion mutants in *A. alternata* (Table 1).

Since the HOG1 MAPK-MAPKK-MAPKKK cascade is genetically intact in the Δnik1 background, it is highly possible that the pathway is associated with another regulatory mechanism to compensate for NIK1 deletion under different developmental or stress conditions. In yeast, the Sho1p transmembrane osmosensor receptor activates the HOG1 MAPK pathway through an alternate MAPKKK, Ste11p (Maeda* et al.*, 1995). It is not known what the putative regulator(s) is and where it relays its signal along the putative HOG1 MAPK pathway in *P. nodorum*. Yoshimi *et al.* (2004) demonstrated that the group III HK Dic1 positively regulates Hog1 signalling in *C. heterostrophus*. However, the HOG1 pathway remained active in the absence of Dic1. Furthermore, a group VI HK SLN1 possesses overlapping roles with HIK1 (group III HK) as upstream regulators of the HOG1 pathway in *M. oryzae* (Jacob* et al.*, 2014; Zhang* et al.*, 2010). blast analysis identified SNOG_11562 as the only group VI HK in *P. nodorum*.

We demonstrated that the Δhog1 mutants possess increased sensitivity to heat stress. The role of the Hog1 pathway in the heat-stress response was first described in *S. cerevisiae* (Winkler* et al.*, 2002). It has been shown that HOG1 is activated during heat stress. In the vascular wilt fungus *Fusarium proliferatum*, conidia of the Δhog1 mutants exposed to heat stress showed reduced viability (Adam* et al.*, 2008b). Therefore, we proposed that heat tolerance is mediated through the HOG1 pathway via an alternate unknown regulator or an alternate pathway independent of Nik1 in *P. nodorum*. Similarly, increased tolerance to MBOA is a phenotype unique to the HOG1 deletion background. The *P. nodorum* necrotrophic effector ToxA induces MBOA production (Du Fall & Solomon, 2013). Although the biochemical target and mode of action of MBOA are yet to be fully deduced, the phytoalexin alters central carbon metabolism in *P. nodorum* (Du Fall & Solomon, 2013). Since HOG1 deletion increases tolerance to MBOA, the mutation presents an ideal tool for functional characterization of resistance. In wheat crown rot fungus, *Fusarium graminearum*, an *N*-malonyltransferase encoded by the FDB2 gene is required for the detoxification of benzoxazolinones (Kettle* et al.*, 2015). However, reciprocal blast analysis indicates that *P. nodorum* does not possess an FDB2 orthologue (Gardiner* et al.*, 2012). Therefore, *P. nodorum* must employ another mechanism for detoxifying MBOA. To our knowledge, this study is a first to examine the effect of a phytoalexin on fungal mutants that are defective in HOG1 signalling.

*P. nodorum* produces fruiting bodies called pycnidia during its asexual state. Pycnidiospores are produced and naturally dispersed via rain splashes to nearby healthy plant tissues to facilitate secondary infection (Eyal* et al.*, 1987; Eyal, 1999). We have demonstrated that NIK1 and HOG1 play a vital role in asexual sporulation. *P. nodorum* Δhog1 mutants, in particular, produced significantly less spores than SN15 and Δnik1 mutants under axenic conditions. This effect is much more pronounced during infection with the Δhog1 mutants where pycnidial production was either abolished at 32 °C or severely reduced at 28 °C. The Δhog1 mutants are generally more susceptible to environmental stresses than SN15 and Δnik1, as demonstrated in this study. A probable explanation is that the Δhog1 mutants may have encountered a combination of stressors during growth on the host plant that is sufficient to antagonize asexual sporulation through the formation of fruiting bodies *in planta* as opposed to favourable conditions presented in axenic cultures. This suggests that HOG1 is required by *P. nodorum* to complete its natural pathogenic lifecycle through asexual sporulation. The molecular mechanism of asexual sporulation in *P. nodorum* is a subject of strong investigations. Previous reverse genetic studies have demonstrated the requirement of signalling pathways, mannitol and trehalose metabolism in asexual sporulation (Gummer* et al.*, 2012; IpCho* et al.*, 2010; Li* et al.*, 2008; Lowe* et al.*, 2009; Solomon* et al.*, 2004, 2005a,  c, 2006b). Deletion of heterotrimeric G-protein-regulated putative short-chain dehydrogenase genes SCH1 and SCH2 resulted in reduced sporulation (Casey* et al.*, 2010; Tan* et al.*, 2008). In the case of SCH1 deletion, pycnidial maturation was blocked (Tan* et al.*, 2008). Intriguingly, sporulation *in vitro* is promoted by the supplementation of γ-aminobutyric acid, a metabolite that accumulates in stressed plants (Mead* et al.*, 2013). It is possible that the reduction in asexual sporulation observed for the Δnik1 and Δhog1 mutants is a pleotropic phenotype resulting from the loss of physiological coordination at the biochemical level.

Inactivation of the HOG1 pathway in many phytopathogenic fungi results in an increase in resistance to dicarboximides and phenylpyrroles (Table 1). In this study, perturbation of HOG1 signalling in *P. nodorum* resulted in increased resistance to a phenylpyrrole and dicarboximide fungicides. Dicarboximides and phenylpyrroles are contact non-systemic site-specific fungicides that are used to control a narrow spectrum of fungal diseases caused by *Botrytis *spp. and the closely related genera *Monilinia *and *Sclerotinia *(Detweiler* et al.*, 1983; Yoshimura* et al.*, 2004). Point mutations in the group III HK accounted for some cases of dicarboximide resistance, observed in field fungal isolates exhibiting low to medium levels of resistance to iprodione. For instance, widespread resistance to iprodione was observed in *B. cinerea* isolated from strawberries (Grabke* et al.*, 2014). Point mutations in the group III HK gene BOS1, efflux pump activity and unknown mechanisms accounted for the different level of resistance observed. Fludioxonil resistance in field fungal isolates is commonly associated with up-regulation of multidrug ABC transporters (Hahn & Leroch, 2015), although laboratory-generated fungal mutants clearly demonstrated the role of Hog1 signalling in resistance (Table 1). The primary target site has not been clearly identified; however, it is thought that these fungicides exert their influence on the group III HKs through the HAMP domain (Fujimura* et al.*, 2015).

The role of the osmotolerance pathway in fungal virulence has been extensive investigated. In many cases, perturbation of the pathway resulted in a drastic reduction or loss of fungal virulence (Table 1). Mutants perturbed in the pathway often demonstrated abnormalities in vegetative morphogenesis, such as abnormal appressoria development (Igbaria* et al.*, 2008), altered fungal cell wall (Jacob* et al.*, 2014), or in ability to form invasive filamentous growth (Mehrabi* et al.*, 2006). These pleiotropic effects may contribute to the loss in pathogenic fitness. In this study, deletions in NIK1 and HOG1 did not affect the ability of *P. nodorum* to cause lesions on wheat; thus, indicating a dispensable role of the pathway in virulence. This is similarly observed in *Bipolaris oryzae, F. proliferatum* and *Colletotrichum lagenarium* deleted in HOG1 (Adam* et al.*, 2008a; Kojima* et al.*, 2004; Moriwaki* et al.*, 2006), and *Sclerotinia sclerotiorum* deleted in group III HK orthologues (Duan* et al.*, 2013). In one instance, deletion of AlHK1 (a NIK1 group III HK orthologue) in the tobacco pathogen *Alternaria longpipes* conferred an increase in virulence (Duan* et al.*, 2013). Thus, the HOG1 pathway plays both overlapping (e.g. osmotolerance) and diverse roles across different pathosystems.

Perturbation of group III HK and components of the Hog1 MAPK pathway often resulted in an increase in sensitivity to oxidative stress (Table 1). However, this was not the case with *P. nodorum* strains deleted in HOG1 and NIK1. Thus, *P. nodorum* uses an alternate pathway to sense and adapt to oxidative stress. It was previously observed that AaHSK1 from *A. alternata* and AbNIK1 from the black spot fungus *Alternaria brassicicola* do not play a role in oxidative stress tolerance (Cho* et al.*, 2009; Lin & Chung, 2010).

Despite the wealth of studies, there is a lack of knowledge of downstream targets associated with the HOG1 pathway in phytopathogenic fungi that could explain phenotypic changes associated with pathway inactivation. In* B. cinerea*, a macroarray approach designed from expressed sequenced tags was used to analyse the gene expression profile of a mutant disrupted in BcSAK1, a HOG1 MAPK gene (Heller* et al.*, 2012). Similar to *P. nodorum* HOG1, BcSAK1 is required for abiotic stress tolerance and conidiation. Interestingly, most genes regulated by BcSAK1 are not associated with stress response. Instead, genes that are associated with the production of phytotoxic secondary metabolites were significantly down-regulated. Furthermore, HOG1 in *F. graminearum* regulates the production of the mycotoxins deoxynivalenol and zealarone (Nyugen* et al.*, 2012). Analysis of the *P. nodorum* genome identified genes encoding 23 polyketide synthases (PKSs), 14 non-ribosomal peptide synthetases (NRPSs), 1 PKS-NRPS hybrid, 3 sesquiterpene synthases and 1 diterpene synthase (Chooi* et al.*, 2014). These genes are often associated in functional gene clusters. This highlights the huge potential for secondary metabolite production in *P. nodorum*. Recent studies have begun to link biologically active secondary metabolites to genes (Chooi* et al.*, 2015a, b; Tan* et al.*, 2009c). It remains to be seen whether the HOG1 pathway regulates secondary metabolism in *P. nodorum* under the appropriate conditions. Therefore, the Δhog1 and Δnik1 mutants generated in this study are ideal tools that can be used to study global regulation. As such, we are currently performing comprehensive analyses of these mutants using RNA sequencing, proteomics, phosphorylation assays and metabolomics to provide a deeper insight to the regulation of HOG1 signalling and its downstream targets. This will allow the dissection of physiological components that contributed to phenotypic impairments, explain differences between both mutants and uncover novel aspects of fungal metabolism in a devastating necrotrophic pathogen of wheat. In the past, these approaches were used to successfully identify downstream (direct and indirect) targets of Fus3/Kss1 MAPK, Stu1 transcription factor and heterotrimeric G-protein signalling in *P. nodorum* (Casey* et al.*, 2010; Gummer* et al.*, 2013; IpCho* et al.*, 2010; Tan, 2007; Tan* et al.*, 2008, 2009a). An establishment of the ‘regulome’ in *P. nodorum* will significantly bolster our understanding of the link between signal transduction and the pathogenic lifecycle.

## Supplementary Data

354 Supplementary File 1
