# Supplementary material for: Dissecting the role of histidine kinase and HOG1 mitogen-activated protein kinase signalling in stress tolerance and pathogenicity of Parastagonospora nodorum on wheat
Source: Microbiology (Reading). 2016 Jun;162(6):1023–36. doi: 10.1099/mic.0.000280 (PMC5042077; doi:10.1099/mic.0.000280)
Supplement: Supplementary file 1 [file mic-162-1023-s001.pdf]

Table S1. Primer sequences.

Primer	Sequence (5'-3')
Nik1qPCRf	TCCTTCCAGATTCTCCTTGC
Nik1qPCRr	CTTCTGAATGTGTTGGACGG
Hog1qPCRf	CTTTGACCCTAAGAAGCGTG
Hog1qPCRr	CATGTGTCAACGGGAAGATC
ActinqPCRf	AGTCGAAGCGTGGTATCCT
ActinqPCRr	ACTTGGGGTTGATGGAG
5_Hog1F	CGCCGTGCCTTGAAACTTGA
5_Hog1R	TGTGACTTTTGGTTACGCCGTCTTCGCAGGATGTGGGGTATGC
3_Hog1F	TCTCCTATGAGTCGTTTACCCAGAAGACGAACCAGTTGCTGAGGAGA
3_Hog1R	AGTCTACGACACACACAAGAGGG
5_Nik1F	GCATTGTGCCACGAGAGTCGT
5_Nik1R	TGTGACTTTTGGTTACGCCGTCTCGCCTTTGCGCCATTTGCAG
3_Nik1F	TCTCCTATGAGTCGTTTACCCAGAACTACCTGTCCAAACCCCTCCG
3_Nik1R	CGCCTTTGCGCCATTTGCAG
Nik1FC	CCCTTGTATCTCTACACACAGGGCTAGATGGTGCTGTATACGATG
Nik1RC	ATGGTGTGGGTTTGGAGACTG
Hog1FC	CCCTTGTATCTCTACACACAGGCTTGAACGTACACTTGCCAGG
Hog1RC	GTCTTAGCCCTCAAGAACGC
pAN8f	AGACGGCGTAACCAAAAGTCACA
pAN8r	TTCTGGGTAAACGACTCATAGGAGA
pAN7fa	AAGTGGAAAGGCTGGTGTGC
pAN7ra	CCTGTGTGTAGAGATACAAGGG
Nik_1F	CGCTAGGTGCCCATGACTAG
Nik_2R	GCAGACCATTCAACATGCCC
Hog_1F	CTACTGCTGCTCTGTCGCAAC
Hog_2R	ACAACCGCCTTTTGTACCG
Ble_1R	GACGCAACGACCTTGTCAAC
Ble_2F	GACGACGTGACCCTGTTCATC

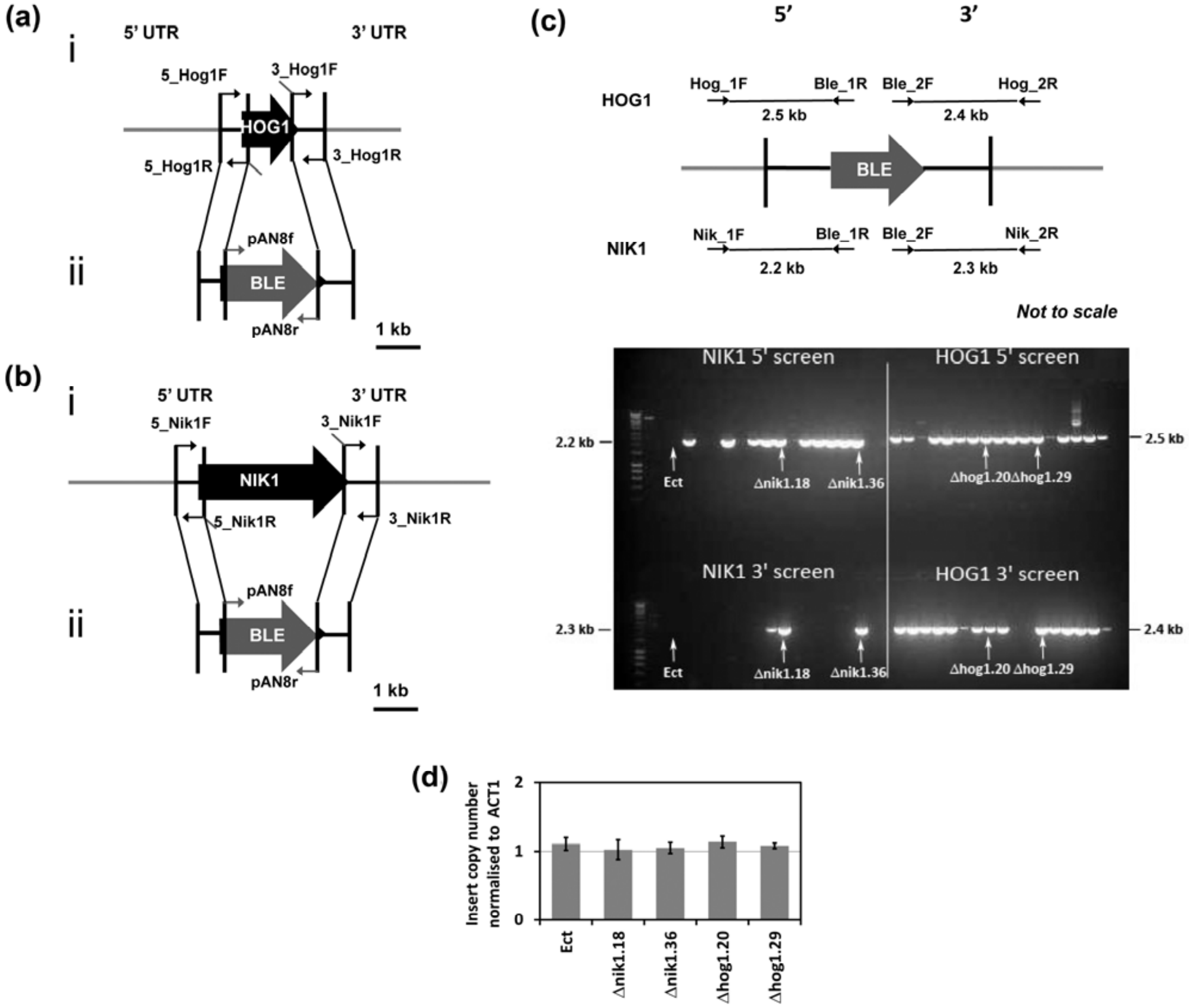

Fig. S1. Construction of the (a) HOG1 and (b) NIK1 knockout vectors. i. 5’ and 3’ UTR of HOG1 and NIK1 were amplified with primers that contain flanking sequences for phleomycin resistance cassette (BLE) from pAN8-1. ii. These PCR fragments were sequentially fused to BLE using fusion PCR. Both resulting vectors were individually transformed into *P. nodorum* SN15. (c) PCR was used to screen transformants for integration of the knockout vector at the targeted site. Correct amplification at the deleted locus indicates successful gene knockout. No amplification indicates ectopic integration elsewhere in the genome. An ectopic strain (Ect) was selected from the NIK1-BLE transformation further analysis. Intact NIK1 and HOG1 in the Ect strain was confirmed by gene specific PCR (data not shown). (d) Copy number of HOG1- and NIK1-BLE knockout cassettes normalised to a single copy of ACT1. Ect is the ectopic control strain. Biological triplicates were used in the copy number assay. Error bars are shown as standard error of the mean.

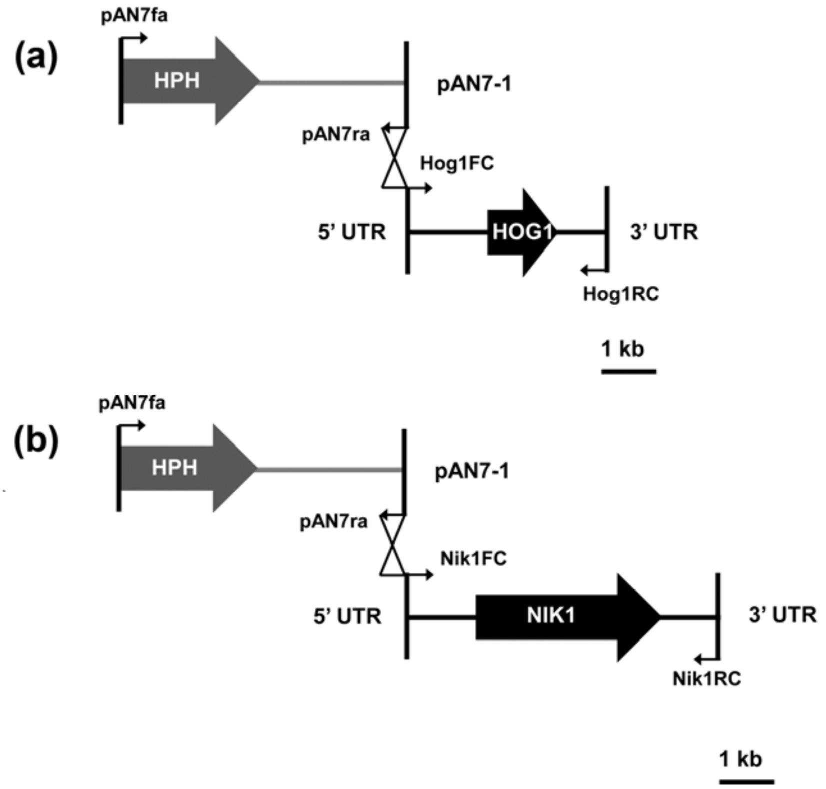

Fig. S2. Genetic complementation of *P. nodorum* Δhog1.20 and Δnik1.18. Construction of the (a) HOG1 and (b) NIK1 gene complementation vectors. HOG1 and NIK1 consisting of 5’ and 3’ UTR regions were amplified with primers that contain flanking sequences for hygromycin resistance cassette (HPH) from pAN7-1. These fragments were fused to HPH using fusion PCR. Both resulting vectors were individually transformed into *P. nodorum* mutants that lacked the respective genes.

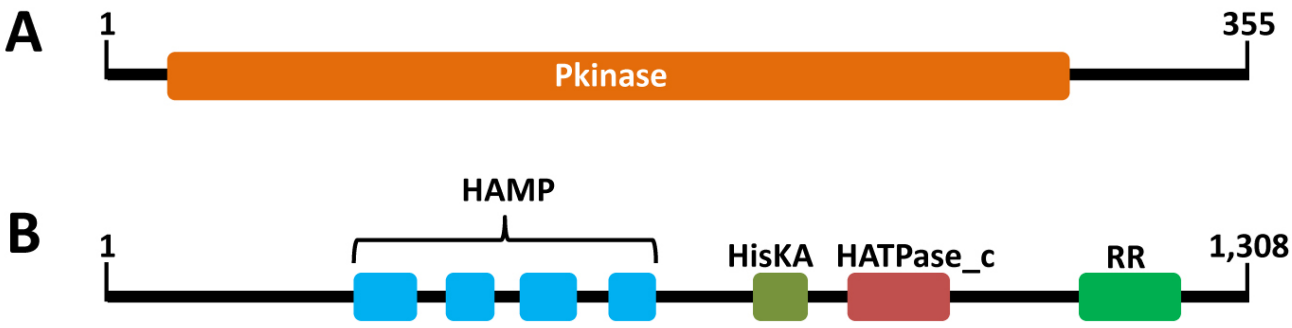

Fig. S3. Scaled schematics of the domain structure for (a) Hog1 and (b) Nik1 from *P. nodorum*. Pkinase – protein kinase domain; HAMP - histidine kinase, adenylyl cyclase, methyl-accepting protein, and phosphatase domain; HisKA – histidine kinase A/phospho-acceptor domain; HATPase_c - histidine kinase-like ATPase; RR – response regulator receiver domain.

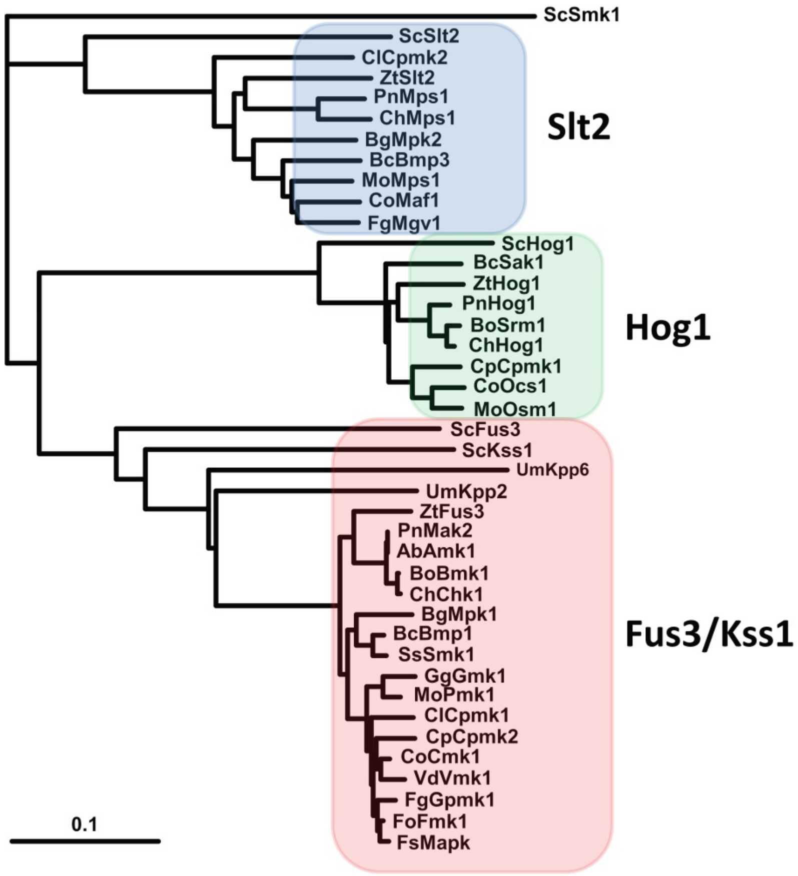

Fig. S4. A phylogram showing amino acid relationship between putative MAPKs of plant pathogenic fungi. ScSmk MAPK from *Saccharomyces cerevisiae* was used as an outgroup. ClustalW2 was used to perform the sequence alignment (Larkin et al., 2007). 'Gonnet' was used as a 'protein weight matrix', 'gap open' of '10', 'gap extension' of '20' and 'gap distances' of '5'. MAPKs are divided into 3 distinct classes according to Xu (2000) based on homology to the yeast MAPK nomenclature. The phylogenetic tree was constructed using TreeView 1.6.6. The bar indicates the relative measure of the distance in the phylogenetic tree as given by TreeView (Page, 1996). Accession details and organisms of all aligned sequences are provided in Supplemental data S1.

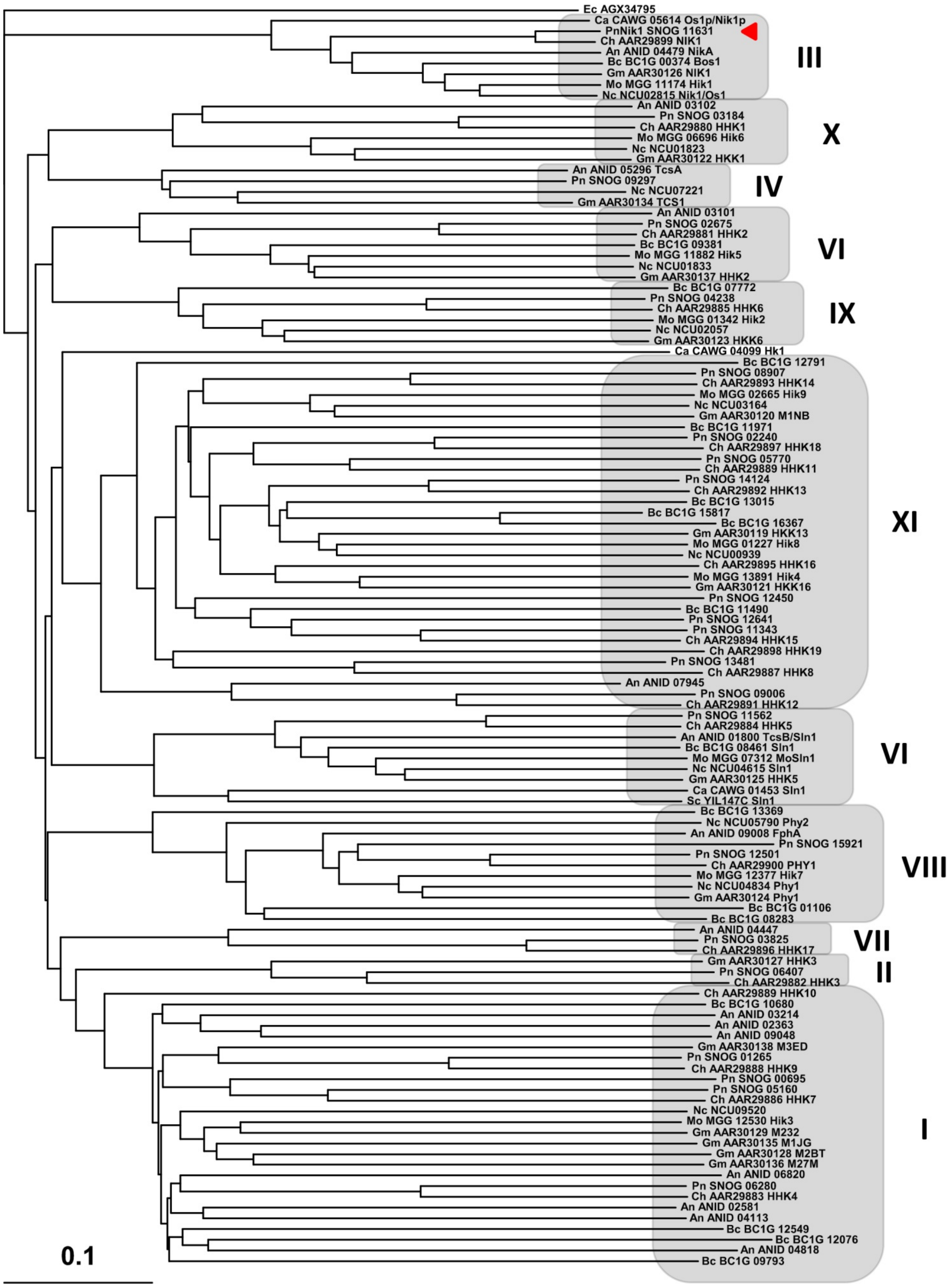

Fig. S5. Continued to the next page.

Fig. S5. Continued from the previous page. A phylogram showing amino acid relationship between *P. nodorum* and other fungal putative hybrid HKs. A hybrid sensory histidine kinase from *E. coli* (Ec_ AGX34795) was used as an outgroup. ClustalW2 was used to perform the sequence alignment (Larkin et al., 2007). 'Gonnet' was used as a 'protein weight matrix', 'gap open' of '10', 'gap extension' of '20' and 'gap distances' of '5'. Hybrid HKs are divided into 11 distinct groups according to Catlett et al. (2003). The phylogenetic tree was constructed using TreeView 1.6.6. The bar indicates the relative measure of the distance in the phylogenetic tree as given by TreeView (Page, 1996). Accession details and organisms of all aligned sequences are provided in Supplemental data S2. Red arrow indicates *P. nodorum* Nik1.

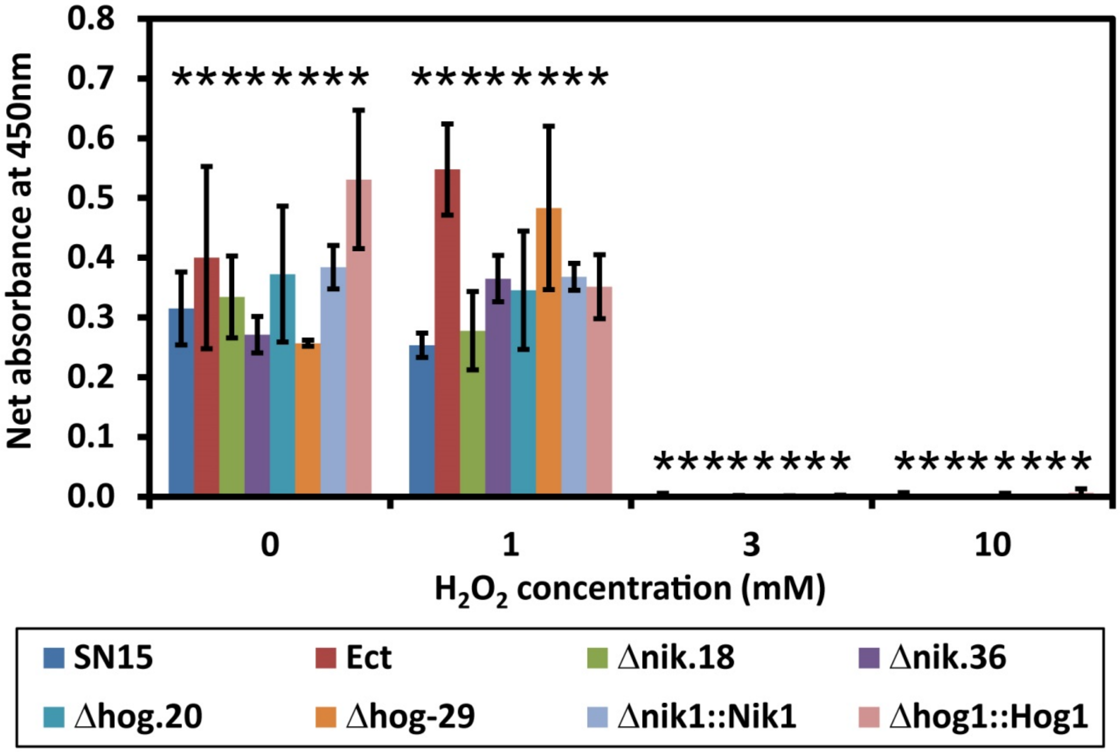

Fig. S6. HOG1 and NIK1 are not required for oxidative stress tolerance. Net absorbance was determined following 5 days growth in MM broth supplemented with different H_2O_2 concentrations using a 96-well plate assay. The experiment performed in four biological replicates. Error bars are shown as standard error of the mean. The Tukey-Kramer test set at a significance threshold of $P \leq 0.05$ was used to compare all measurements at each H_2O_2 treatment. The number of '*' above bars indicate significant differences between strains.

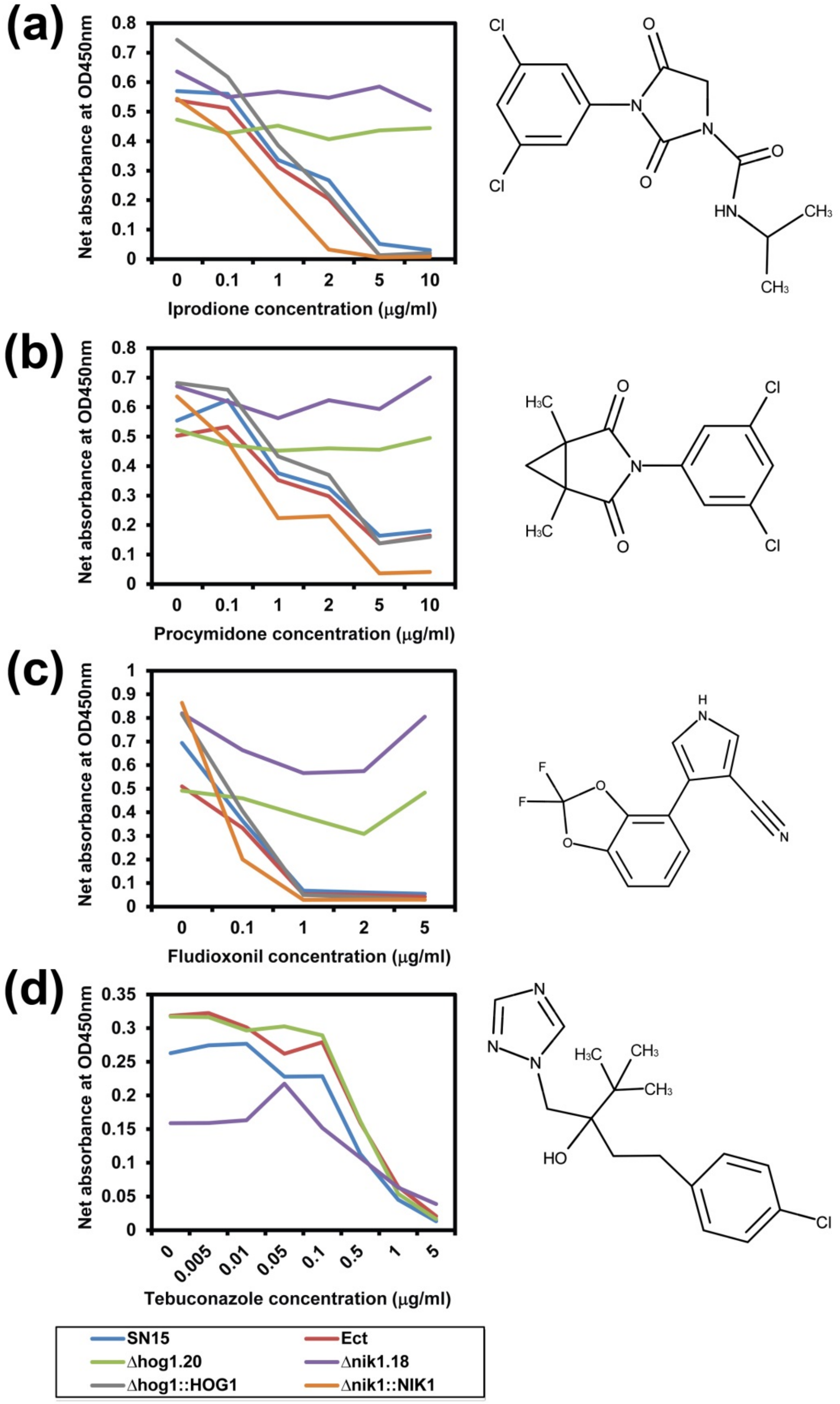

Fig. S7. Assessing *P. nodorum* growth in fungicides using a 96-well microtitre plate assay. Average absorbance difference (representing growth) of each strain following 6 days growth in minimal medium broth supplemented with different concentrations of (a) iprodione (dicarboximide), (b) procymidone (dicarboximide), (c) fludioxonil (phenylpyrrole) and (d) tebuconazole (triazole). Chemical structures of all fungicides are shown to the right. Marvin Live (ChemAxon, Hungary) was used to draw all chemical structures. EC50 and RF values are described in Table 3.

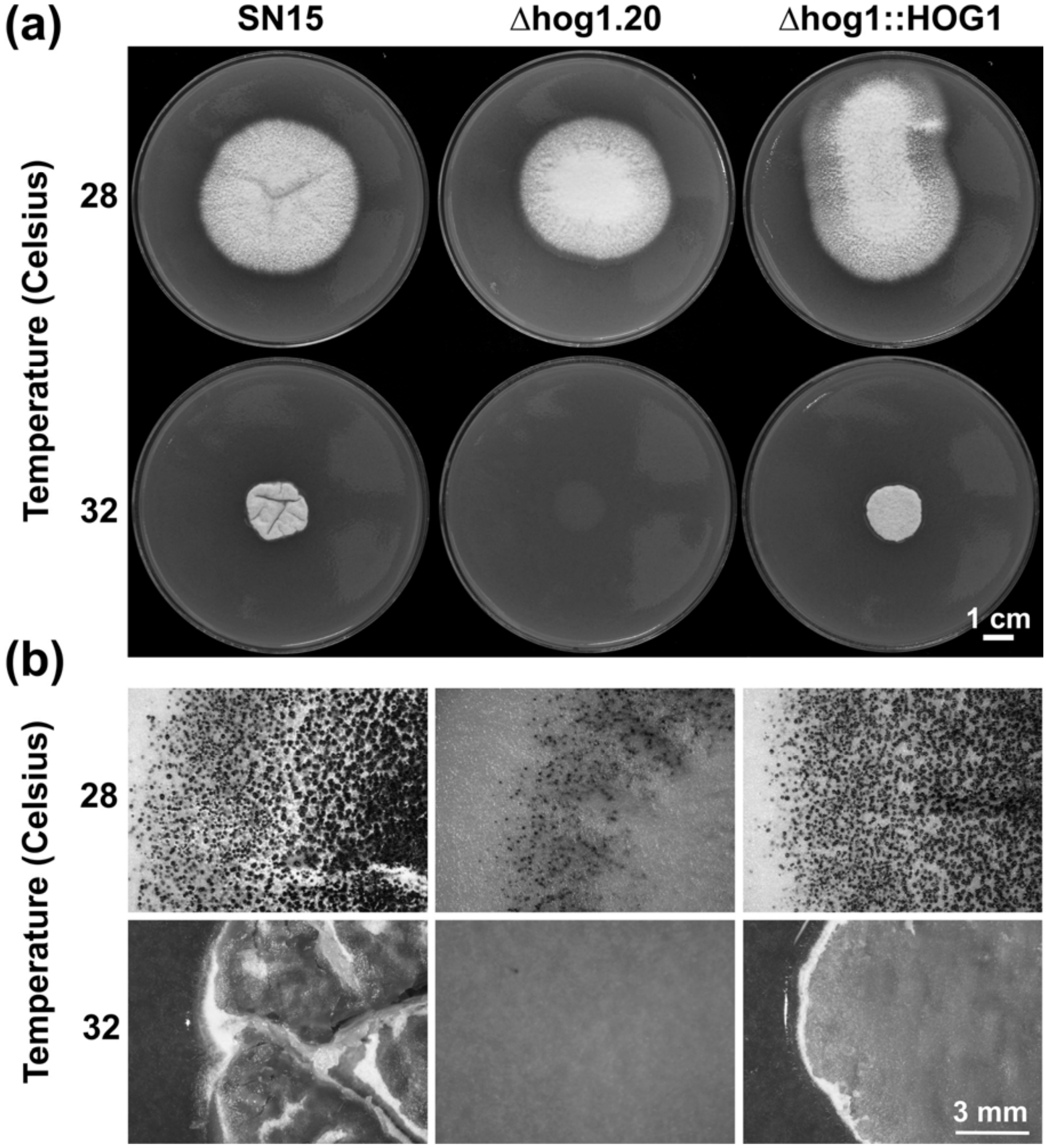

Fig. S8. Genetic complementation of *P. nodorum* Δhog1.20 (Δhog1::HOG1) with HOG1 restored wild-type (a) vegetative growth and (b) pycnidiation under temperature stress *in vitro*.

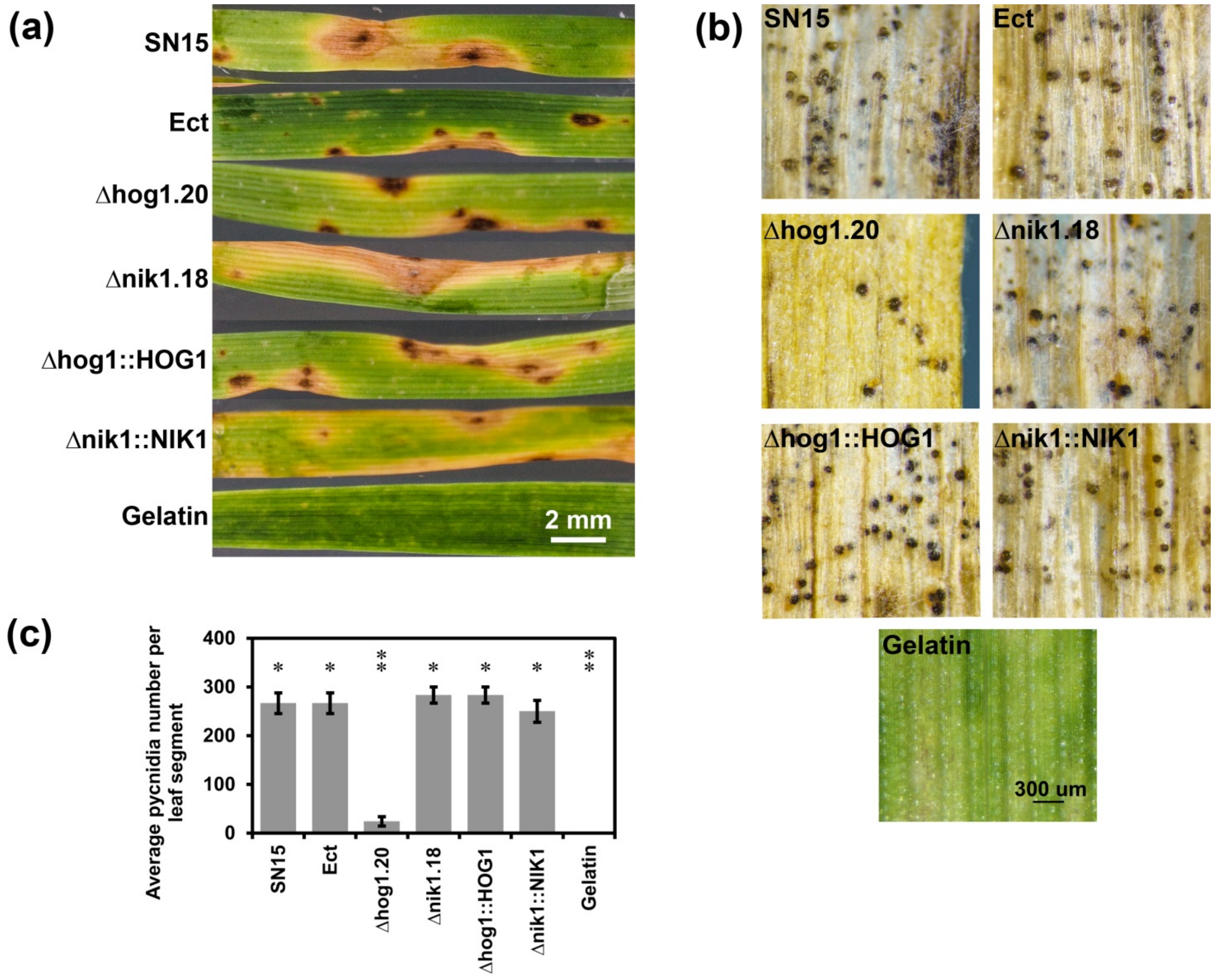

Fig. S9. Wheat infection assay at 28°C. (a) Necrotic lesions caused by SN15, Ect, Δnik1.18, Δhog1.20 and genetically complemented strains. Gelatin was used as a non-infection treatment. (b) Pycnidia were allowed to develop on a detached leaf assay. (c) Average pycnidia count of infected leaf segments. An analysis of variance using the Tukey-Kramer test set at a significance threshold of $P \leq 0.05$ was used to compare the pycnidiation capability of all strains. Error bars are shown as standard error of the mean. The number of '*' above bars indicate significant differences between strains. The experiment was performed with six biological replicates.

Supplemental data S1. Accession details for amino acid sequences of fungal MAPKs. All P. nodorum amino acid sequences were obtained from https://github.com/robsyme/Parastagonospora_nodorum_SN15. All other sequences were obtained from NCBI Genbank.

ScFus3_Saccharomyces_cerevisiae_Acc:P16892
ScKss1_Saccharomyces_cerevisiae_Acc:AAZ22456
ScSlt2_Saccharomyces_cerevisiae_Acc:Q00772
ScHog1_Saccharomyces_cerevisiae_Acc:P32485
ScSmk1_Saccharomyces_cerevisiae_Acc:P41808
PnMak2_Parastagonospora_nodorum_Acc:AAX63387
PnMps1_Parastagonospora_nodorum_(SNOG_05764)
AbAmk1_Alternaria_brassicicola_Acc:AAS20192
BoBmk1_Bipolaris_oryzae_Acc:BAD42855
BoSrm1_Bipolaris_oryzae_Acc:BAE48722
BgMpk1_Blumeria_graminis_Acc:AAG53654
BgMpk2_Blumeria_graminis_Acc:AAG53655
BcBmp1_Botrytis_cinerea_Acc:AAG23132
BcBmp3_Botrytis_cinerea_Acc:ABJ51957
BcSak1_Botrytis_cinerea_Acc:CAJ85638
ClCpmk1_Claviceps_purpurea_Acc:CAC47939
ClCpmk2_Claviceps_purpurea_Acc:CAC87145
ChChk1 Cochliobolus heterostrophus Acc:AAF05913
ChMps1 Cochliobolus heterostrophus Acc:ABM54149
ChHog1 Cochliobolus heterostrophus Acc:BAD99295
CoMaf1 Colletotrichum lagenarium Acc:AAL50116
CoOcs1 Colletotrichum lagenarium Acc:BAD11137
CoCmk1 Colletotrichum lagenarium Acc:AAD50496
CpCpmk1 Cryphonectria parasitica Acc:AAO27796
CpCpmk2 Cryphonectria prasitica Acc:AAP86959
FgGpmk1 Fusarium graminearum Acc:AF448230
FgMgv1 Fusarium graminearum Acc:AF492766
FoFmk1 Fusarium oxysporum Acc:AAG01162
GgGmk1 Gaeumannomyces graminis Acc:AAG44657
FsMapk Fusarium solani Acc:Q00859
MoPmk1 Magnaporthe oryzae Acc:AAC49521
MoMps1 Magnaporthe oryzae Acc:AAC63682
MoOsm1 Magnaporthe oryzae Acc:AAF09475
ZtFus3 Zymoseptoria tritici Acc:AAX81518
ZtSlt2 Zymoseptoria tritici Acc:AAY98511
ZtHog1 Zymoseptoria tritici Acc:ABD92790
UmKpp2 Ustilago maydis Acc:AAF15528
UmKpp6 Ustilago maydis Acc:CAD43731
VdVmk1 Verticillium dahliae Acc:AAW71477
SsSmk1 Sclerotinia sclerotiorum Acc:AAQ54908
PnHog1_Parastagonospora_nodorum_Acc:Q0U4L8

Supplemental data S2. Accession details for amino acid sequences of fungal hybrid HKs. All of *P. nodorum* amino acid sequences were obtained from https://github.com/robsyme/Parastagonospora_nodorum_SN15; *Saccharomyces cerevisiae* from http://www.yeastgenome.org/; *Botrytis cinerea*, *Candida albicans*, *Aspergillus nidulans*, *Neurospora crassa* and *Magnaporthe oryzae* from http://www.broadinstitute.org/scientific-community/data; *Cochliobolus heterostrophus* and *Gibberella moniliformis* from http://www.ncbi.nlm.nih.gov/genbank/. Continued on the next page.

Parastagonospora_nodorum_Pn(SNOG_01265)
Parastagonospora_nodorum_Pn(SNOG_02675)
Parastagonospora_nodorum_Pn(SNOG_03184)
Parastagonospora_nodorum_Pn(SNOG_03825)
Parastagonospora_nodorum_Pn(SNOG_08907)
Parastagonospora_nodorum_Pn(SNOG_11343)
Parastagonospora_nodorum_Pn(SNOG_11562)
Parastagonospora_nodorum_Pn(SNOG_12501)
Parastagonospora_nodorum_Pn(SNOG_09297)
Parastagonospora_nodorum_Pn(SNOG_12641)
Parastagonospora_nodorum_Pn(SNOG_12450)
Parastagonospora_nodorum_Pn(SNOG_02240)
Parastagonospora_nodorum_Pn(SNOG_04238)
Parastagonospora_nodorum_Pn(SNOG_05160)
Parastagonospora_nodorum_Pn(SNOG_05770)
Parastagonospora_nodorum_Pn(SNOG_06280)
Parastagonospora_nodorum_Pn(SNOG_06407)
Parastagonospora_nodorum_Pn(SNOG_09006)
Parastagonospora_nodorum_PnNik1(SNOG_11631)
Parastagonospora_nodorum_Pn(SNOG_14124)
Parastagonospora_nodorum_Pn(SNOG_15921)
Parastagonospora_nodorum_Pn(SNOG_00695)
Parastagonospora_nodorum_Pn(SNOG_13481)
Ecoli_Ec(AGX34795)
Botrytis_cinerea_Bc(BC1G_11971)
Botrytis_cinerea_Bc(BC1G_13015)
Botrytis_cinerea_Bc(BC1G_12076)
Botrytis_cinerea_Bc(BC1G_01106)
Botrytis_cinerea_Bc(BC1G_13369)
Botrytis_cinerea_Bc(BC1G_08283)
Botrytis_cinerea_Bc(BC1G_00374_Bos1)
Botrytis_cinerea_Bc(BC1G_09381)
Botrytis_cinerea_Bc(BC1G_11490)
Botrytis_cinerea_Bc(BC1G_12549)
Botrytis_cinerea_Bc(BC1G_08461_Sln1)
Botrytis_cinerea_Bc(BC1G_15817)
Botrytis_cinerea_Bc(BC1G_12791)
Botrytis_cinerea_Bc(BC1G_10680)
Botrytis_cinerea_Bc(BC1G_07772)

Supplemental data S2. Continued on the next page.
Botrytis_cinerea_Bc(BC1G_09793)
Botrytis_cinerea_Bc(BC1G_16367)
Candida_albicans_Ca(CAWG_05614_Os1p/Nik1p)
Candida_albicans_Ca(CAWG_04099_Hk1)
Candida_albicans_Ca(CAWG_01453_Sln1)
Aspergillus_nidulans_An(ANID_04479_NikA)
Aspergillus_nidulans_An(ANID_05296_TcsA)
Aspergillus_nidulans_An(ANID_01800_TcsB/Sln1)
Aspergillus_nidulans_An(ANID_02581)
Aspergillus_nidulans_An(ANID_02363)
Aspergillus_nidulans_An(ANID_03101)
Aspergillus_nidulans_An(ANID_03102)
Aspergillus_nidulans_An(ANID_03214)
Aspergillus_nidulans_An(ANID_04113)
Aspergillus_nidulans_An(ANID_04447)
Aspergillus_nidulans_An(ANID_04818)
Aspergillus_nidulans_An(ANID_06820)
Aspergillus_nidulans_An(ANID_07945)
Aspergillus_nidulans_An(ANID_09048)
Aspergillus_nidulans_An(ANID_09008_FphA)
Magnaporthe_oryzae_Mo(MGG_07312_MoSln1)
Magnaporthe_oryzae_Mo(MGG_11174_Hik1)
Magnaporthe_oryzae_Mo(MGG_01342_Hik2)
Magnaporthe_oryzae_Mo(MGG_12530_Hik3)
Magnaporthe_oryzae_Mo(MGG_13891_Hik4)
Magnaporthe_oryzae_Mo(MGG_11882_Hik5)
Magnaporthe_oryzae_Mo(MGG_06696_Hik6)
Magnaporthe_oryzae_Mo(MGG_12377_Hik7)
Magnaporthe_oryzae_Mo(MGG_01227_Hik8)
Magnaporthe_oryzae_Mo(MGG_02665_Hik9)
Neurospora_crassa_Nc(NCU02815_Nik1/Os1)
Neurospora_crassa_Nc(NCU01823)
Neurospora_crassa_Nc(NCU01833)
Neurospora_crassa_Nc(NCU00939)
Neurospora_crassa_Nc(NCU02057)
Neurospora_crassa_Nc(NCU03164)
Neurospora_crassa_Nc(NCU04615_Sln1)
Neurospora_crassa_Nc(NCU04834_Phy1)
Neurospora_crassa_Nc(NCU05790_Phy2)
Neurospora_crassa_Nc(NCU07221)
Neurospora_crassa_Nc(NCU09520)
Saccharomyces_cerevisiae_Sc(YIL147C_Sln1)
Cochliobolus_heterostrophus_Ch(AAR29880_HHK1)
Cochliobolus_heterostrophus_Ch(AAR29881_HHK2)
Cochliobolus_heterostrophus_Ch(AAR29882_HHK3)

Supplemental data S2. Continued from the previous page.
Cochliobolus_heterostrophus_Ch(AAR29883_HHK4)
Cochliobolus_heterostrophus_Ch(AAR29884_HHK5)
Cochliobolus_heterostrophus_Ch(AAR29885_HHK6)
Cochliobolus_heterostrophus_Ch(AAR29886_HHK7)
Cochliobolus_heterostrophus_Ch(AAR29887_HHK8)
Cochliobolus_heterostrophus_Ch(AAR29888_HHK9)
Cochliobolus_heterostrophus_Ch(AAR29889_HHK10)
Cochliobolus_heterostrophus_Ch(AAR29889_HHK11)
Cochliobolus_heterostrophus_Ch(AAR29891_HHK12)
Cochliobolus_heterostrophus_Ch(AAR29892_HHK13)
Cochliobolus_heterostrophus_Ch(AAR29893_HHK14)
Cochliobolus_heterostrophus_Ch(AAR29894_HHK15)
Cochliobolus_heterostrophus_Ch(AAR29895_HHK16)
Cochliobolus_heterostrophus_Ch(AAR29896_HHK17)
Cochliobolus_heterostrophus_Ch(AAR29897_HHK18)
Cochliobolus_heterostrophus_Ch(AAR29898_HHK19)
Cochliobolus_heterostrophus_Ch(AAR29899_NIK1)
Cochliobolus_heterostrophus_Ch(AAR29900_PHY1)
Gibberella_moniliformis_Gm(AAR30119_HKK13)
Gibberella_moniliformis_Gm(AAR30120_M1NB)
Gibberella_moniliformis_Gm(AAR30121_HKK16)
Gibberella_moniliformis_Gm(AAR30122_HKK1)
Gibberella_moniliformis_Gm(AAR30123_HKK6)
Gibberella_moniliformis_Gm(AAR30124_Phy1)
Gibberella_moniliformis_Gm(AAR30125_HHK5)
Gibberella_moniliformis_Gm(AAR30126_NIK1)
Gibberella_moniliformis_Gm(AAR30127_HHK3)
Gibberella_moniliformis_Gm(AAR30128_M2BT)
Gibberella_moniliformis_Gm(AAR30129_M232)
Gibberella_moniliformis_Gm(AAR30134_TCS1)
Gibberella_moniliformis_Gm(AAR30135_M1JG)
Gibberella_moniliformis_Gm(AAR30136_M27M)
Gibberella_moniliformis_Gm(AAR30137_HHK2)
Gibberella_moniliformis_Gm(AAR30138_M3ED)
